# Dipeptidyl peptidase-4 inhibitors and GLP-1 reduce myocardial infarct size in a glucose-dependent manner

**DOI:** 10.1186/1475-2840-12-154

**Published:** 2013-10-22

**Authors:** Derek J Hausenloy, Hannah J Whittington, Abigail M Wynne, Shah S Begum, Louise Theodorou, Niels Riksen, Mihaela M Mocanu, Derek M Yellon

**Affiliations:** 1 The Hatter Cardiovascular Institute, UCL Institute of Cardiovascular Science and NIHR University College London Hospitals Biomedical Research Centre, 67 Chenies Mews, London WC1E 6HX, UK; 2 Department of General Internal Medicine, Radboud University Nijmegen Medical Centre, Nijmegen, the Netherlands

**Keywords:** Ischaemia, Reperfusion, Glucagon-like peptide 1, Sitagliptin, Vildagliptin, Dipeptidyl peptidase-4 inhibitor, Blood glucose

## Abstract

**Background:**

The dipeptidyl peptidase-4 (DPP-4) inhibitors Sitagliptin and Vildagliptin lower blood glucose by augmenting endogenous levels of glucagon-like peptide-1 (GLP-1), an incretin which also confers cardioprotection. As such, we hypothesized that treatment with DPP-4 inhibitors are also cardioprotective.

**Methods:**

In *ex vivo* experiments: Male Sprague–Dawley rats were randomized to receive by oral gavage either Vildagliptin (20 mg/kg/day), Sitagliptin (100 mg/kg/day), or water for 2 weeks. Excised hearts were Langendorff-perfused with buffer containing either 5 mmol/L or 11 mmol/L glucose and subjected to 35 minutes ischaemia/120 minutes reperfusion. In *in vivo* experiments: Male young Wistar and Sprague–Dawley rats, middle aged Wistar and Goto-Kakizaki diabetic rats were randomized to receive by oral gavage either Sitagliptin (100 mg/kg/day), or water for 2 weeks. Rats were then subjected to 30 minutes ischaemia/120 minutes reperfusion and infarct size ascertained.

**Results:**

Two weeks pre-treatment with either Vildagliptin or Sitagliptin reduced *ex vivo* myocardial infarction (MI) size in hearts perfused with buffer containing 11 mmol/L glucose but not 5 mmol/L glucose. This effect was abolished by Exendin 9–39 (GLP-1 receptor antagonist) and H-89 (PKA antagonist). Treatment of perfused hearts with native GLP-1 was also glucose-sensitive, reducing MI size, at glucose concentrations 7, 9, and 11 mmol/L but not at 5 mmol/L. Finally, Sitagliptin reduced *in vivo* MI size in middle aged Wistar (7-8 mmol/L glucose) and Goto-Kakizaki (9-10 mmol/L glucose) rats where blood glucose was elevated, but not in young Wistar (5 mmol/L glucose) or Sprague–Dawley (5 mmol/L glucose) rats, where blood glucose was normal.

**Conclusions:**

We find that chronic treatment with DPP-4 inhibitors reduced MI size, via the GLP-1 receptor-PKA pathway, in a glucose-dependent manner. Glucose-sensitive cardioprotection of endogenous GLP-1 in diabetic patients may in part explain why intensive control of serum glucose levels has been associated with increased cardiovascular risk.

## Background

Coronary heart disease (CHD) is the leading cause of death in diabetic patients. According to the World Health Organisation more than 220 million people worldwide have diabetes. Patients with diabetes are two to three times more likely to develop CHD, and experience worse clinical outcomes following an acute myocardial infarction [1-3], coronary angioplasty [4], and cardiac bypass surgery [5-7]. The reason for the worse cardiovascular outcomes in diabetic patients is currently unclear. Pre-clinical animal studies suggest that there may be specific defects in diabetic cardiomyocytes which may be responsible including increased mitochondrial generation of reactive oxygen species, impaired signalling via the PI3-Akt survival kinase pathway and reduced rates of ATP synthesis (reviewed in [8,9]). Clearly, novel cardioprotective strategies are required to improve clinical outcomes in diabetic patients with CHD.

In this respect, emerging evidence suggests that the incretin, glucagon-like peptide (GLP-1), GLP-1(7–36)amide and its breakdown product GLP-1(9–36)amide both have the ability to protect the heart against acute ischaemia-reperfusion injury (IRI) [10-12], as well as having direct beneficial effects on metabolism [13,14]. However, GLP-1 is rapidly broken down in the circulation by the enzyme, dipeptidyl peptidase-4 (DPP-4). The anti-diabetic agents, Sitagliptin and Vildagliptin both lower blood glucose by inhibiting DPP-4, which augments endogenous levels of GLP-1 [15]. Therefore, in this study we utilized both *ex vivo* and *in vivo* models of ischaemia reperfusion injury (IRI) to investigate whether chronic treatment with the DPP-4 inhibitors, Sitagliptin and Vildagliptin, also confer cardioprotection.

## Methods

### Animals

Animal experiments were conducted in strict accordance with the Animals (Scientific Procedures) Act 1986 published by the UK Home Office and the Guide for the Care and Use of Laboratory Animals published by the US National Institutes of Health (NIH Publication No. 85–23, revised 1996). Approval has been granted by the University College London ethics review board. All efforts were made to minimize suffering.

Male Sprague–Dawley (SD) rats (3-4 months) were used for the *ex vivo* isolated heart investigations. Male SD rats (3–4 months), Wistar rats (3–4 months), middle aged Wistar rats (7–8 months) and middle aged Goto Kakizaki (GK) rats (7–8 months) were used for the *in vivo* experiments. Animals received humane care in accordance with the United Kingdom Animal (Scientific Procedures) Act of 1986. Approval was granted by a university ethics review board. The study conforms with the Guide for the Care and Use of Laboratory Animals published by the US National Institutes of Health (NIH Publication No. 85–23, revised 1996). For oral gavage, Sitagliptin (100 mg/kg/day) and Vildagliptin (20 mg/kg/day) were dissolved in water, concentrations sufficient to increase GLP-1 levels [16,17]. All other reagents were of standard analytical grade.

### Ex vivo isolated perfused rat heart model of acute myocardial infarction

Rats were terminally anesthetised with sodium pentobarbital (55 mg/kg intraperitoneally) and heparin (300 IU). The hearts were rapidly excised into ice-cold buffer, and mounted on a constant pressure (80 mmHg) Langendorff-perfusion apparatus and perfused with modified Krebs-Henseleit bicarbonate buffer in mmol/L: NaCl 118.5, NaHCO_3_ 25.0, KCl 4.8, MgSO_4_ 1.2, KH_2_PO_4_ 1.2, CaCl_2_ 1.7 and glucose 5.0 or 11.0. The buffer was gassed with 95%O_2_/ 5%CO_2_ and pH maintained at 7.35-7.45 at 37.0°C. A suture was placed around the left main coronary artery and the ends inserted into a pipette tip to form a snare. A latex, fluid-filled balloon was placed in the left ventricle through an incision in the left atrial appendage and inflated to a pressure of 8–10 mmHg. Left ventricular developed pressure, heart rate and coronary flow were monitored at regular intervals. Temperature was constantly measured via a thermo-probe inserted into the pulmonary artery and maintained between 37.0 ± 0.2°C. Regional myocardial ischaemia was induced by tightening the suture placed around the left anterior descending coronary artery (LAD) for 35 minutes and reperfusion for 120 minutes initiated by releasing the snare. At the end of the reperfusion period the suture was tied and the heart perfused with 0.25% Evans Blue in saline to delineate the area at risk. Hearts were frozen at -20°C for several hours before infarct size determination.

### In vivo rat model of acute myocardial infarction

Rats were anesthetised with sodium pentobarbital (20 mg/kg intraperitoneally) and heparin (300 IU). The rats were intubated and ventilated with a Harvard ventilator (room air, 70 strokes/min, tidal volume: 8-9 ml/kg). Body temperature was maintained at 37.4 ± 1°C by means of a rectal probe thermometer attached to a temperature control system (CMA450). A lateral thoracotomy was performed to expose the heart and a suture placed around the LAD. The suture was tightened using a loop system to create LAD ligation and regional ischaemia which was confirmed by a change in ECG profile. Following 30 minutes of ischaemia, the vessel was reperfused for 120 minutes. At the end of reperfusion, the heart was removed from the chest, the LAD permanently occluded and the heart perfused with 0.5% Evans blue in saline to delineate the area at risk. Hearts were frozen at -20°C for several hours before infarct size determination.

### Myocardial Infarct size determination

All hearts were sliced into 2 mm thick transverse sections and incubated in triphenyltetrazolium chloride solution (TTC; 1% in phosphate buffer). TTC reacts with intracellular dehydrogenases to stain viable risk zone tissue red leaving the infarcted areas off-white. The slices were then transferred and fixed in 10% formalin overnight. All the slices from one heart were scanned into the computer for analysis or drawn onto acetate, and the area at risk, area of infarction, and area of viable risk zone was delineated and converted to a volume assuming a 2 mm slice thickness. Image J or computerised planimetry (Summa Sketch III, Summagraphics, Seymour, CT, USA) were then used to assess the percentage of infarcted tissue in the myocardium area at risk (I/R%) [18].

### Blood glucose assessment

Samples for fasting blood glucose were taken at baseline and after 2 weeks treatment with Sitagliptin, Vildagliptin or water control. Blood glucose measurements (mmol/L) were determined using an ABL 700 series blood gas analyzer (Radiometer, Copenhagen).

### Experimental protocols for isolated heart perfusion

To investigate the effect of different glucose concentrations within the perfusion buffer, isolated rat hearts were perfused with 5 mmol/L versus 11 mmol/L glucose during control and ischaemic preconditioned (IPC) experiments (the latter used as a positive control). Hearts were perfused with buffer containing glucose at either 5 mmol/L or 11 mmol/L and subjected to stabilization, ischaemia and reperfusion or to a standard IPC protocol (2 cycles of 5 minutes global ischaemia and 10 minutes reperfusion) prior to ischaemia and reperfusion.

**Figure 1:**
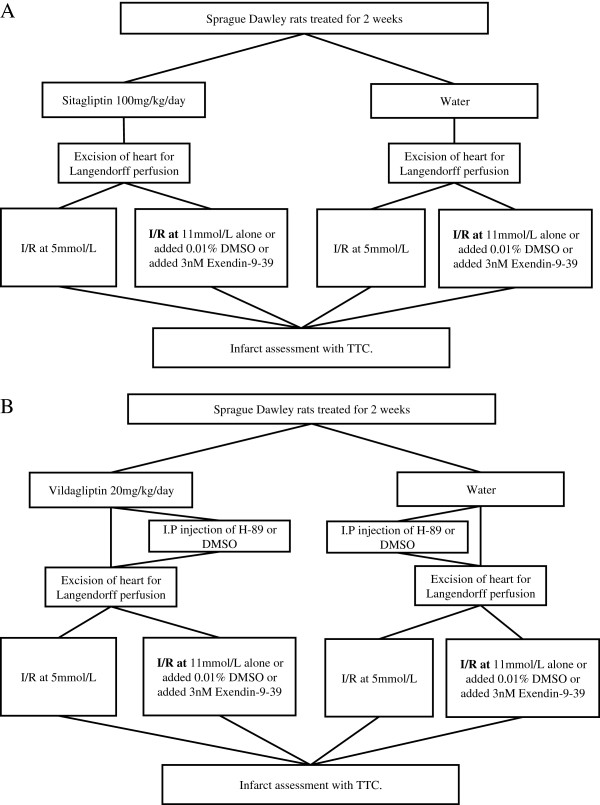
**Ex-vivo experimental schedule for Sitagliptin and Vildagliptin pre-treatment. (A)** Sprague–Dawley rats were treated for 2 weeks Sitagliptin (100 mg/kg/day) or water. **(B)** Sprague–Dawley rats were treated for 2 weeks Vildagliptin (20 mg/kg/day) or water. All hearts were subjected to 35 minutes ischaemia followed by 120 minutes reperfusion (I/R).

Animals were randomly assigned to receive, Sitagliptin (100 mg/kg/day), Vildagliptin (20 mg/kg/day) or water alone given daily by oral gavage for 2 weeks. Hearts were then excised from animals and mounted on the Langendorff-apparatus, and randomized to receive one of the experimental protocols summarized in Figures 1A and 1B. To examine the role of GLP-1 and PKA in the setting of the anti-diabetic gliptin drugs, Exendin 9–39 or DMSO (3 nM, a GLP-1 receptor antagonist, Sigma) were added into the perfusate in selected experiments and H-89 or DMSO (5 μM, a PKA antagonist, Sigma) was administered by intraperitoneal (I.P) injection prior to excision of the heart.

To investigate the cardioprotective effects of GLP-1 (0.3 nM, Novo Nordisk, Denmark) at different concentrations of glucose; hearts were rapidly excised from untreated rats and mounted on the Langendorff-apparatus. The perfusion buffer contained 5, 7, 9 or 11 mmol/L of glucose; and either DMSO vehicle (0.01%) or GLP-1 (0.3 nM) throughout the entire protocol.

### Experimental protocols for in vivo investigations

To investigate whether the cardioprotective effect of DPP-4 inhibition were glucose sensitive in the *in vivo* setting; rats with differing blood glucose levels were treated by oral gavage with either Sitagliptin (100 mg/kg/day) or water for 2 weeks prior to LAD occlusion/reperfusion. Four groups were assessed SD, Wistar, middle aged Wistar and middle aged GK rats.

### Statistical analysis

All values are expressed as mean ± SEM. Myocardial infarct size was analyzed by one-way ANOVA and Fisher’s protected least significant difference test for multiple comparisons. Differences were considered significant when P < 0.05.

## Results

### Both Sitagliptin and Vildagliptin pre-treatment reduced myocardial infarct size ex vivo in a glucose-dependent manner

There was no difference in the area at risk or haemodynamic variables between the treatment groups. The blood glucose levels after 2 week of treatment averaged 5.5 ± 0.3 mmol/L, 5.7 ± 0.2 mmol/L and 5.7 ± 0.4 mmol/L for Sitagliptin, Vildagliptin and control, respectively.

**Figure 2:**
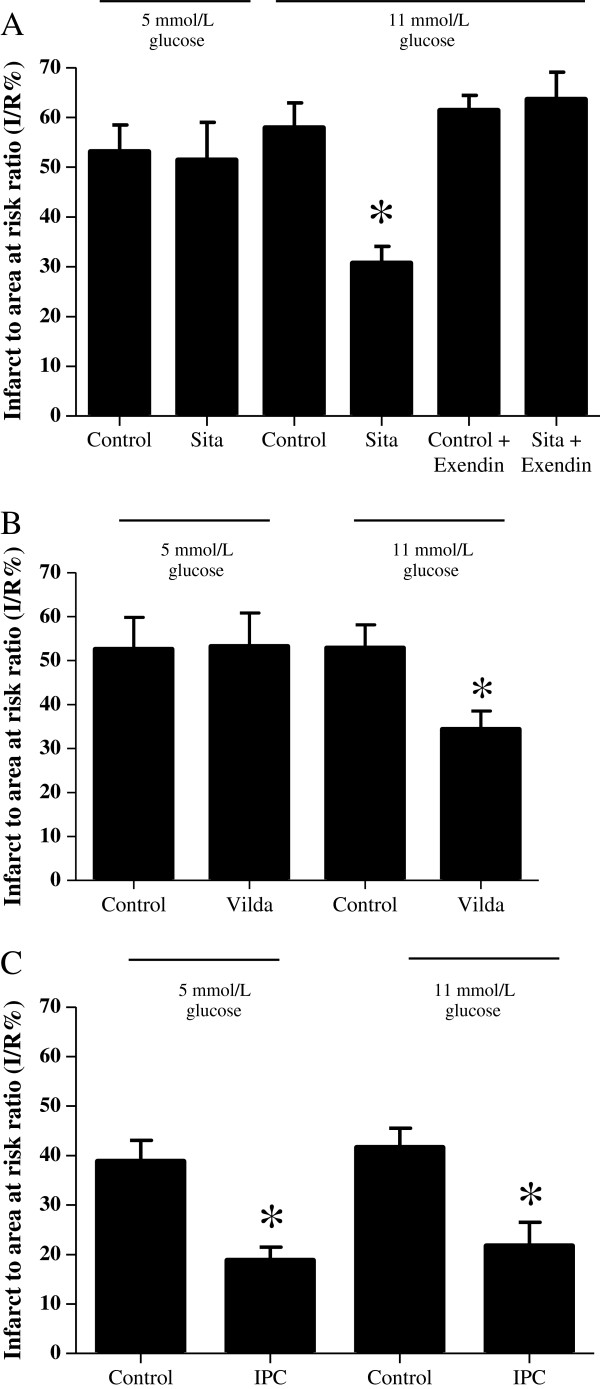
**Ex-vivo myocardial infarct size following Sitagliptin or Vildagliptin pre-treatment. (A)** Ex vivo myocardial infarct size expressed as a percentage of the risk zone. A. Pre-treatment with Sitagliptin (Sita) reduced the myocardial infarct to area at risk ratio (I/R%) in isolated rat hearts perfused with buffer containing 11 but not 5 mmol/L glucose. This infarct-limiting effect was abolished in hearts Langendorff-perfused with Exendin 9–39 (Exendin), a GLP-1 receptor antagonist. *P < 0.05. N ≥ 6/group. **(B)** Pre-treatment with Vildagliptin (Vilda) reduced the myocardial infarct to area at risk ratio (I/R%) in isolated rat hearts perfused with buffer containing 11 but not 5 mmol/L glucose. *P < 0.05. N ≥ 6/group. **(C)** Ischaemic preconditioning (IPC) reduces the myocardial infarct to area at risk ratio (I/R%) in isolated rat hearts perfused with buffer containing either 5 or 11 mmol/L glucose. *P < 0.05. N ≥ 6/group.

Sitagliptin pre-treatment was found to reduce myocardial infarct size in hearts perfused with buffer containing 11 mmol/L of glucose (30.7 ± 3.4% with Sitagliptin versus 57.9 ± 5.0% with control: P < 0.05:N ≥ 6/group) but not 5 mmol/L glucose (51.4 ± 7.6% with Sitagliptin versus 53.1 ± 5.4% with control: P > 0.05:N ≥ 6/group) (Figure 2A). Similarly, Vildagliptin pre-treatment reduced myocardial infarct size in hearts perfused with buffer containing 11 mmol/L of glucose (34.4 ± 4.1% with Vildagliptin versus 52.9 ± 5.2% with control: P < 0.05:N ≥ 6/group) but not 5 mmol/L glucose (53.2 ± 4.8% with Vildagliptin versus 52.6 ± 7.2% with control: P > 0.05:N ≥ 6/group) (Figure 2B). Interestingly, ischaemic preconditioning (IPC) (used as a positive control in this setting) reduced myocardial infarct size in perfused rat hearts at both 5 mmol/L glucose (18.8 ± 2.7% with IPC versus 38.8 ± 4.3% with control: P < 0.05: N > 6/group) and 11 mmol/L glucose (21.7 ± 4.8% with IPC versus 41.6 ± 3.9% with control: P < 0.05: N ≥ 6/group) (Figure 2C).

### Pharmacological inhibition of the GLP-1 receptor and the PKA signalling pathway abrogated both Sitagliptin and Vildagliptin-induced cardioprotection

**Figure 3:**
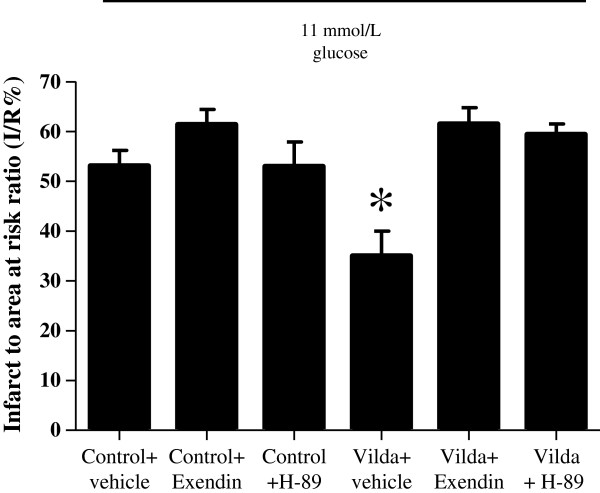
**Ex vivo myocardial infarct size expressed as a percentage of the risk zone.** The reduction in myocardial infarct to area at risk ratio (I/R%) induced by pre-treatment with Vildagliptin (Vilda) was abolished by treatment with either Exendin 9–39 (Exendin), a GLP-1 receptor antagonist, or H-89 (a PKA antagonist). *P < 0.05. N ≥ 6/group.

There was no difference in the area at risk or haemodynamic variables between the treatment groups. The infarct-limiting effects of Sitaglipitin at 11 mmol/L glucose were abolished by Exendin 9–39 (a GLP-1 receptor antagonist) (63.6 ± 5.5% with Sitagliptin + Exendin 9–39 versus 61.4 ± 3.0% with Control + Exendin 9–39: P > 0.05:N > 7/group)(Figure 2A) and the cardioprotective effects of Vildagliptin were abolished in the presence of Exendin 9–39 and H-89 (a PKA inhibitor) (61.5 ± 3.3% with Vildagliptin + Exendin 9–39 and 59.4 ± 2.1% with Vildagliptin + H-89 versus 35.0 ± 5.0% with Vildagliptin + vehicle: P < 0.05:N ≥ 6/group)(Figure 3). Importantly, the pharmacological inhibitors themselves did not influence infarct size significantly (61.4 ± 3.0% with control + Exendin 9–39 and 53.0 ± 4.9% with control + H-89 versus 53.1 ± 3.1% with control + vehicle: P > 0.05: N ≥ 6/group) (Figure 3).

### The infarct-limiting effects of GLP-1 treatment are also glucose-sensitive

**Figure 4:**
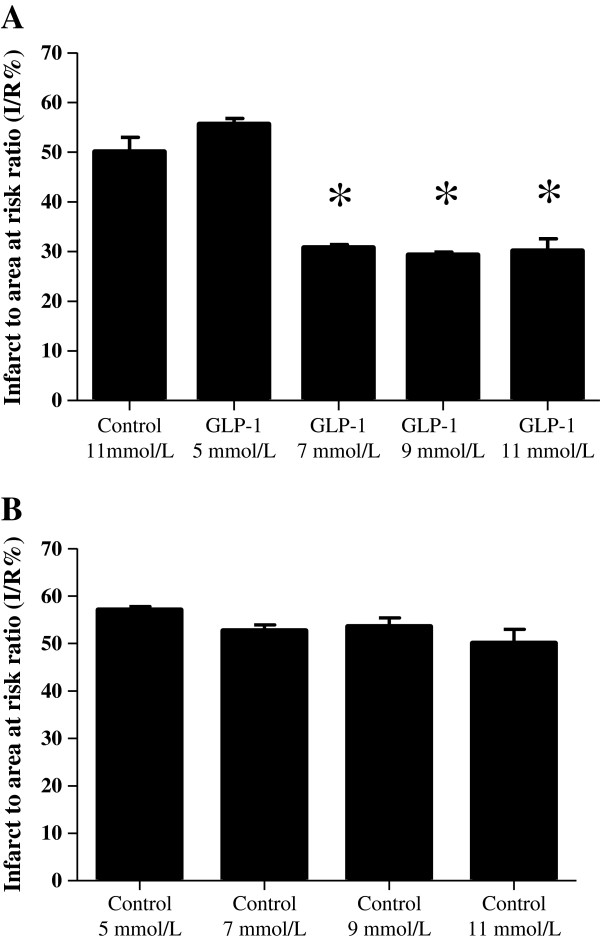
**Ex-vivo myocardial infarct size expressed as a percentage of the risk zone. (A)** There was no difference in myocardial infarct to area at risk ratio (I/R%) in hearts perfused with buffer containing glucose at either 5, 7, 9, or 11 mmol/L. *P < 0.05. N ≥ 6/group. **(B)** Perfusion of isolated rat hearts with buffer containing GLP-1 reduced myocardial infarct to area at risk ratio (I/R%) in hearts perfused with buffer containing glucose at 7, 9, or 11 mmol/L but not at 5 mmol/L. *P < 0.05. N ≥ 6/group.

There was no difference in the area at risk or haemodynamic variables between the treatment groups. The perfusion of hearts with buffer containing GLP-1 reduced MI size, at glucose concentrations 7, 9, and 11 mmol/L but not at 5 mmol/L (30.8 ± 0.6% at 7 mmol, 29.3 ± 0.6% at 9 mmol/L, 39.1 ± 2.5% at 11 mmol/Lpretreatment did not reduce blood glucose levels in any of the groups. versus 55.6 ± 1.2% at 5 mmol/L:P < 0.05:N ≥ 6/group) (Figure 4A). There was no difference in MI size in control hearts perfused at the different glucose concentrations (57.1 ± 0.7% at 5 mmol/L, 52.7 ± 1.2% at 7 mmol/L, 55.6 ± 1.5% at 9 mmol/L, 50.1 ± 2.9% at 11 mmol/L:P > 0.05:N ≥ 6/group)(Figure 4B).

### Sitagliptin pre-treatment reduced myocardial infarct size in vivo in a glucose-dependent manner

Blood glucose levels were not significantly different between SD and Wistar rats, but were significantly different between these groups and middle aged Wistar and GK rats (in mmol/L: 5.5 ± 0.3, 5.6 ± 0.2, 7.5 ± 0.3 and 9.3 ± 1.5, respectively. P < 0.05:N ≥ 6/group). Sitagliptin pre-treatment did not reduce blood glucose levels in any of the groups.

**Figure 5:**
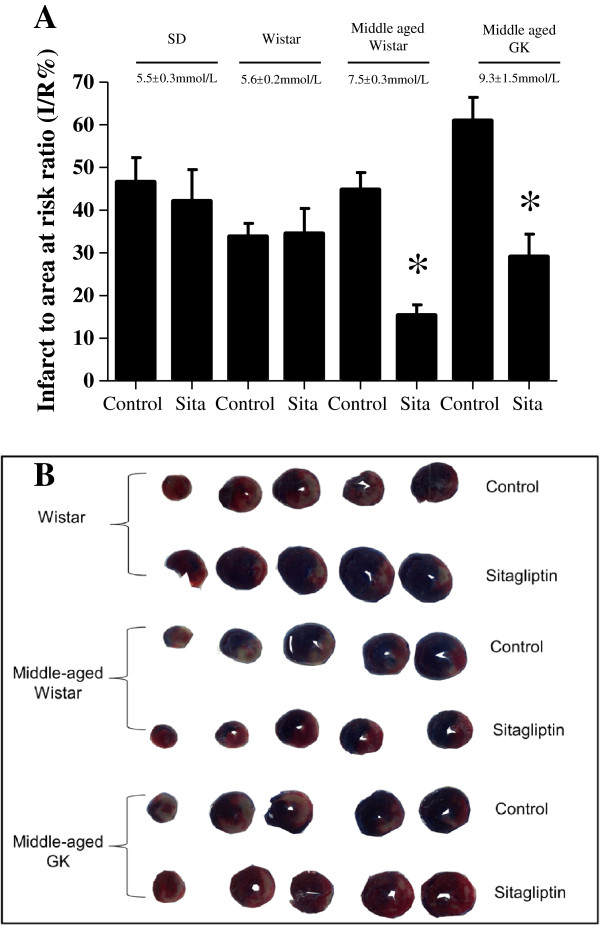
**Effect of Sitagliptin pre-treatment on *in vivo *myocardial infarct size. (A)** Myocardial infarct size is expressed as a percentage of the risk zone. Sitagliptin pre-treatment reduced myocardial infarct to area at risk ratio (I/R%) in middle aged Wistar and GK rats. There was no difference in myocardial infarct to area at risk ratio in SD or Wistar rats. * P < 0.05:N ≥ 6/group. **(B)** Representative short axis slices of control and treated hearts depicting non-ischemic zone (blue), area of myocardial infarction (white), and non-infarcted viable myocardium in the area at risk (red).

In the *in vivo* LAD occlusion/reperfusion studies, Sitagliptin pre-treatment was found to reduce myocardial infarct size in both middle aged Wistar and GK rats (15.4 ± 2.4% with Sitagliptin versus 44.8 ± 4.0% with control in Wistars; and 29.10 ± 5.3% with Sitagliptin versus 60.9 ± 5.5% with control in GKs. P < 0.05:N ≥ 6/group). However, in the SD or Wistar groups there were no differences in MI size (42.1 ± 7.4% and 34.5 ± 5.9% with Sitagliptin versus 46.6 ± 5.7% and 33.8 ± 3.1% with control, respectively for SD and Wistar) (Figure 5 A,B).

## Discussion

The main findings from the present study are as follows: (a) We report that chronic oral treatment with the DPP-4 inhibitors, Sitagliptin or Vildagliptin, is cardioprotective as evidenced by a significant reduction in myocardial infarct size in the isolated perfused rat heart; (b) Interestingly, we show that for the first time, the infarct-limiting effects of Sitagliptin and Vildagliptin pre-treatment are sensitive to the glucose levels present during the myocardial infarction, such that cardioprotection was only observed in hearts infarcted in the presence of a high glucose of 11 mmol/L, with loss of cardioprotection at lower glucose levels of 5 mmol/L; (c) The infarct-limiting effects of DPP-4 inhibitors were abrogated by pharmacological inhibitors of the GLP-1 receptor and the PKA signalling pathway; (d) The cardioprotection elicited by GLP-1 treatment was also glucose-sensitive with infarct limitation observed at glucose levels of 7, 9 and 11 mmol/L but not at 5 mmol/L;(e) Sitagliptin related cardioprotection is also dependent on circulating glucose levels in the *in vivo* setting.

### GLP-1 cardioprotection

Previous experimental studies have demonstrated that exogenous GLP-1 is cardioprotective. These cardioprotective effects were attributed to GLP-1 receptor activation and subsequent recruitment of numerous intracellular signalling pathways involving Akt, Erk1/2, p70S6K, and AMPK as well as the downstream phosphorylation and inhibition of the pro-apoptotic protein BAD (reviewed in [10]). In the current study we were keen to investigate whether endogenous GLP-1 levels, which are augmented by DPP-4 inhibitor therapy, also confer cardioprotection. Whilst conducting the current studies, Ye and co-workers [19] reported infarct-limitation with chronic Sitagliptin therapy through the activation of PKA. Interestingly, in that particular study the average blood glucose was about 7.5 mmol/L, which corresponds to the levels of glucose in which we observed cardioprotection in our study. GLP-1 has also been suggested to improve myocardial contractility. GLP-1 enhanced cardiac voltage-gated L-type Ca^2+^ currents I(_Ca_) in canine ventricular cardiomyocytes. Interestingly, this augmentation was linked to the activation of cAMP-dependent PKA [20].

In contrast, Sauve and co-workers [21] failed to demonstrate myocardial infarct limitation with either genetic or pharmacological inhibition of DPP-4 using DPP-4 knockout mice and Sitagliptin, respectively, even though the blood glucose level was at 10 mmol/L. Finally, it was also demonstrated that pre-treatment with Vildagliptin did not reduce MI size or prevent adverse LV remodelling in rats subjected to MI [22]. However, it is worth noting that in all these studies, these results were obained following permanent LAD ligation, and not a reperfusion MI model and in fact in two of these studies, DPP-4 inhibition exhibited improved survival rates. Interestingly, Zhao et al. [23] also demonstrated that exogenous GLP-1 was able to improve the recovery of left ventricular function in isolated Langendorff-perfused rat hearts subjected to acute IRI at 5 mM glucose. However, in contrast to our study, the low-flow global myocardial ischaemia model was used making a direct comparison of results difficult.

### Other effects of DPP-4 inhibition

Of note, an earlier experimental study attributed the cardioprotective effects of DPP-4 inhibition to the augmentation of endogenous stromal derived factor-1 (SDF-1), a known chemokine substrate of DPP-4 [24]. This latter study illustrates the non-specificity of DPP-4 inhibition for GLP-1, as it has a number of substrates including other gastrointestinal hormones, neuropeptides, cytokines, and chemokines [25]. In this regard, it was important for us to demonstrate that the DPP-4 inhibitor induced infarct-limitation in the isolated heart was blocked by GLP-1 receptor and PKA antagonists, suggesting the involvement of the myocardial GLP-1 receptor and subsequent activation of PKA. Furthermore, the observed glucose- sensitivity was also reproduced *in vivo* in animal models and with GLP-1, providing confirmatory evidence of the mediatory role of endogenous GLP-1 in DPP-4 inhibitor cardioprotection.

### Glucose-dependent cardioprotection

Of interest, our study is the first to report infarct limitation with chronic Sitagliptin and Vildagliptin therapy being glucose-dependent, such that infarct-limitation was observed at a glucose level of 11 mmol/L and not at 5 mmol/L. Interestingly, the glucose-dependency was also observed in GLP-1 mediated cardioprotection with infarct-limitation being observed at glucose levels of 7, 9, and 11 mmol/L but not at 5 mmol/L. Moreover, the finding that Sitagliptin pre-treatment in middle aged Wistar and GK diabetic rats, both with hyperglycaemic blood glucose levels, limited infarct size but these effects were lost in normoglycaemic SD and Wistar rats, strengthens the idea that glucose-dependent cardioprotection with these agents is in fact an interesting phenomenon. It was important for us to demonstrate that the positive control for cardioprotection, ischaemic preconditioning, was still effective in hearts perfused with either 5 or 11 mmol/L, demonstrating that infarct-limitation was possible at the lower blood glucose level in our model. The reason for the glucose-dependency of the cardioprotection elicited by both DPP-4 inhibitors and GLP-1 is not known and further studies are clearly required to investigate the mechanism underlying this observation.

Interestingly, it has been suggested that GLP-1 does not lower blood glucose at levels lower than 5 mM and therefore the risk of hypoglycemia is significantly reduced. Previous experimental studies have reported glucose-dependent changes in intracellular calcium and GLP-1 receptor-PKA signalling favouring high blood glucose levels [26]. Alternatively, the GLP-1 receptor could be differentially regulated in response to circulating blood glucose levels. In the presence of high glucose, GLP-1 could possibly activate G-protein coupled receptor signalling via its G_i_ signalling pathway involving the Akt component of the RISK pathway leading to cardioprotection [27,28], whereas in conditions of low blood glucose this cardioprotective pathway is not favoured. Chai et al. [29] have recently showed that the administration of GLP-1 increased Akt-phosphorylation in cultured cells. However, the link between GLP-1, high glucose and Akt phosphorylation is yet to be fully investigated. Recent evidence has implicated AMPK as a mediator of GLP-1 cardioprotection [30], and low glucose levels have been shown to activate AMPK [31,32], and so the modulation of AMPK may explain in part the glucose-sensitivity of GLP-1 cardioprotection.

### Study limitations

There are several limitations to the current experimental study: (1) Because we only reperfused the hearts for 2 hours in our *in vivo* model of acute IRI, we do not know if the cardioprotective effect elicited by the DPP-4 inhibitors had a long-term infarct-limiting effect: (2) The findings presented here concerning the glucose-sensitivity of GLP-1 cardioprotection are purely descriptive. Clearly further studies are required to elucidate the potential mechanisms underlying the glucose-sensitivity; (3) We only investigated the effect of H-89 (a PKA inhibitor) on Vildagliptin-induced cardioprotection and ideally we should have also shown that H-89 blocked Sitagliptin mediated cardioprotection; (4) We did not measure serum levels of GLP-1.

### Clinical implications

Although, proof-of-concept clinical studies have demonstrated cardioprotective benefit with exogenous GLP-1 therapy in the clinical settings of acute myocardial infarction [33], coronary artery bypass graft surgery [34] and heart failure [35], this treatment regimen requires a chronic subcutaneous infusion of GLP-1 given that the GLP-1 is rapidly broken down in the body by the enzyme DPP-4. Therefore, the findings of the current study suggest that augmenting endogenous levels of GLP-1 using clinically available DPP-4 inhibitors such as Sitagliptin and Vildagliptin may provide for a therapeutic strategy, which not only treats the diabetes but also protects the heart from ischaemia-reperfusion injury, the major cardiac complication of diabetes. An alternative strategy to DPP-4 inhibition would be to administer clinically available GLP-1 analogues such as Liraglutide [36] and Exenatide [37,38] which are resistant to DPP-4 and are also cardioprotective in animal studies. Clinical studies are already underway investigating both GLP-1 analogues (Pharmacological Postconditioning to Reduce Infarct Size Following Primary PCI [POSTCON II]: ClinicalTrials.gov Identifier NCT00835848 and Effect of Additional Treatment With EXenatide in Patients With an Acute Myocardial Infarction (the EXAMI Trial: ClinicalTrials.gov Identifier NCT01254123) and DPP-4 inhibitors [39,40] as therapy for cardiovascular disease. Lonborg et al. [41] has shown that the GLP-1 analogue, Exenatide, could reduce MI size when administered prior to PPCI in non-diabetic STEMI patients when compared to control. In that particular study the blood glucose ranged from 5–8 mmol/L. It would be interesting to know whether the cardioprotective effect of Exenatide was affected by the presenting blood glucose levels.

## Conclusions

Our present study confirms that DPP-4 inhibitors elicit cardioprotection against acute IRI similar to that seen for GLP-1, moreover these effects appear to be glucose-sensitive.

## Abbreviations

DPP-4: Dipeptidyl peptidase-4; GLP-1: Glucagon-like peptide-1; MI: Myocardial infarction; CHD: Coronary heart disease; IRI: Ischaemia reperfusion injury; SD: Sprague–Dawley; GK: Goto Kakizaki; LAD: Left anterior descending coronary artery; TTC: Triphenyltetrazolium chloride; I/R%: Percentage of infarcted tissue in the myocardium area at risk; IPC: Ischaemic preconditioning; SDF-1: Stromal derived factor-1; PKA: Protein kinase A.

## Competing interests

The authors declare that they have no competing interests.

## Authors’ contributions

DMY, DJH, MMM, RDC conceived the studies, wrote, reviewed and edited manuscript. HJW, AMW, SSB, LT, NR performed experiments, wrote, reviewed and edited manuscript. All authors read and approved the final manuscript.
